# Supplementary material for: Dermatillomania: Strategies for Developing Protective Biomaterials/Cloth
Source: Pharmaceutics. 2021 Mar 5;13(3):341. doi: 10.3390/pharmaceutics13030341 (PMC8001957; doi:10.3390/pharmaceutics13030341)
Supplement: Supplementary file 1 [file pharmaceutics-13-00341-s001.pdf]

pharmaceutics

Supplementary Materials: Dermatillomania: Strategies for developing Protective Biomaterials/Cloth

Ravipati Priusha, Bice Conti, Enrica Chiesa and Karine Andrieux

1. Evaluating the Need for Better Treatment Strategies

A. **Online survey on "Understanding Body Focused Repetitive Behaviors (BFRBs)".** A google form based online survey was started with an aim to understand more about BFRBs and to understand the requirement of a better treatment strategy. This survey was conducted to get inputs directly from the individuals with BFRBs on the need for new strategies.

B. **Method. Ethics:** The motive of the survey was explained as follows "You are invited to participate in a survey on "Understanding Body Focused Repetitive Behaviours". In this survey, people will be asked to complete a survey about BFRB. It will take approximately 3–4 minutes to complete the questionnaire. Your participation in this study is completely voluntary. There are no foreseeable risks associated with this project. The whole purpose of this survey is to understand the intensity of the impact of BFRB on the lives of people. However, if you feel uncomfortable answering any questions, you can withdraw from the survey at any point. It is very important for us to learn your opinions. Your survey responses will be strictly confidential and data from this survey will be reported only in the aggregate. Your information will be coded and will remain confidential. Thank you very much for your time and support." This was followed by the question "We require you to give us your consent as a participant of this survey and that nobody is forcefully asking you to take this survey". Only individuals who gave their consent were allowed to be a part of the survey.

This survey included questions related to body focussed repetitive behaviours (BFRBs). The survey link was posted in two closed Facebook groups related to BFRBs on 1st March 2020 and was kept open for responses until 30th April 2020. These two Facebook groups were chosen with an assumption that individuals joined these private groups in order to share their experiences or stories of living with BFRBs and to seek community support from other individuals with the same struggles.

2. Results

A total of 112 respondents have given their consent to be a part of this survey. The first 61 responses were excluded from being analysed as Question "what kind of BFRB you have?" did not show the option Dermatillomania due to a technical glitch which was later corrected, so the remaining 51 responses were analysed that were received post correction.

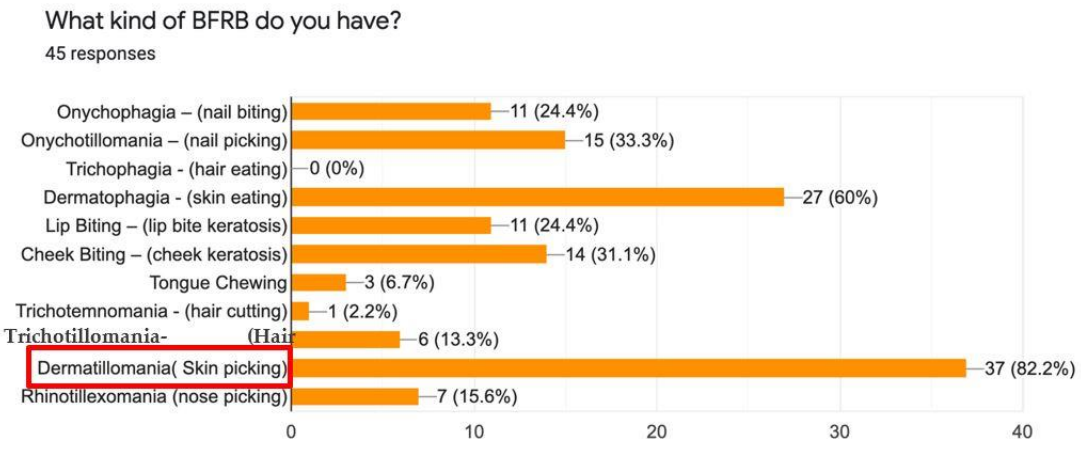

Figure S1. Incidence of body focused repetitive behaviours**.**

About 88.2% ($N = 45$) out of 51 self-reported having Dermatillomania or Dermatophagia or both along with other comorbid BFRBs. Out of the 45 individuals which consisted 91.1% ($n = 41$) of females, 17.7% ($n = 8$) reported having Dermatophagia, 40% ($n = 18$) reported having Dermatillomania and 42.2% ($n = 19$) reported having both Dermatillomania and Dermatophagia. In total 82.2% ($n = 37$) of the 45 individuals self-reported having Dermatillomania. These individuals also reported having other BFRBs.

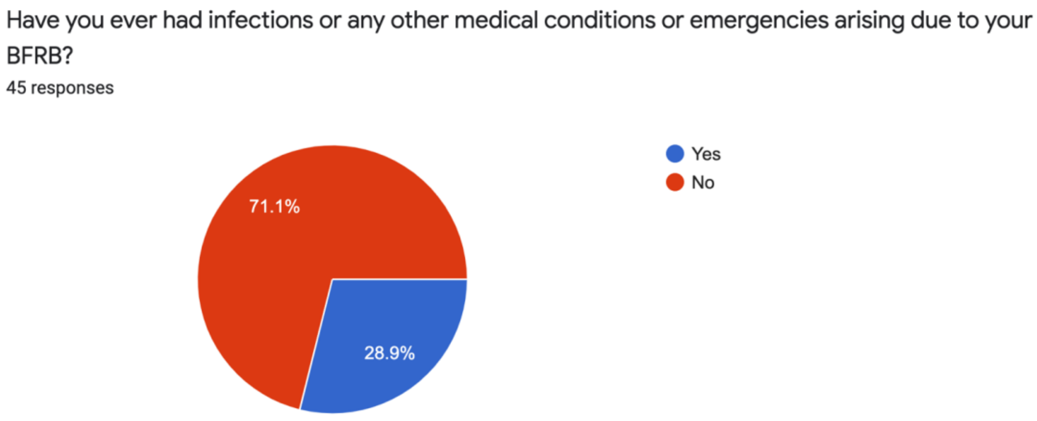

Figure S2. Occurrence of infections due to skin picking behaviour.

About 28.9% ($n = 13$) of 45 individuals reported having infections or medical complications due to picking behaviour.

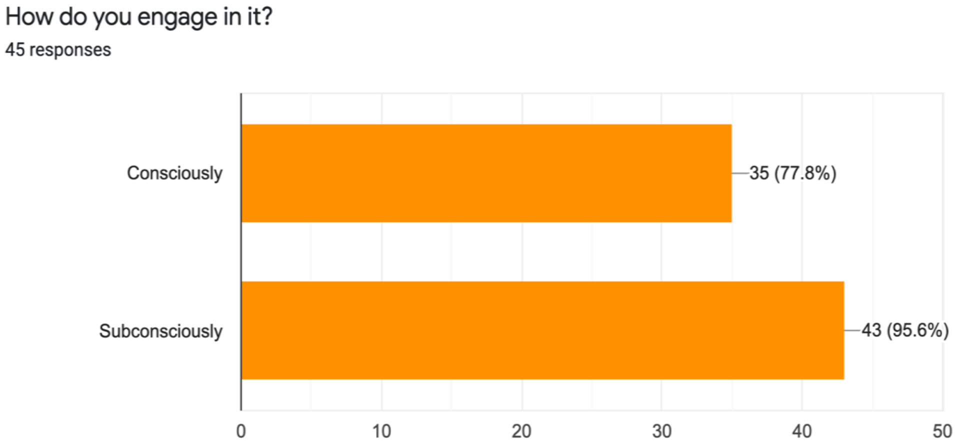

Figure S3. Mode of engagement in skin picking behaviour.

About 73.3% (*N* = 33) out of 45 reported engaging in behaviour both consciously and subconsciously while 22.2% (*n* = 10) reported engaging subconsciously and 4.4% (*n* = 2) reported engaging consciously in the behaviour.

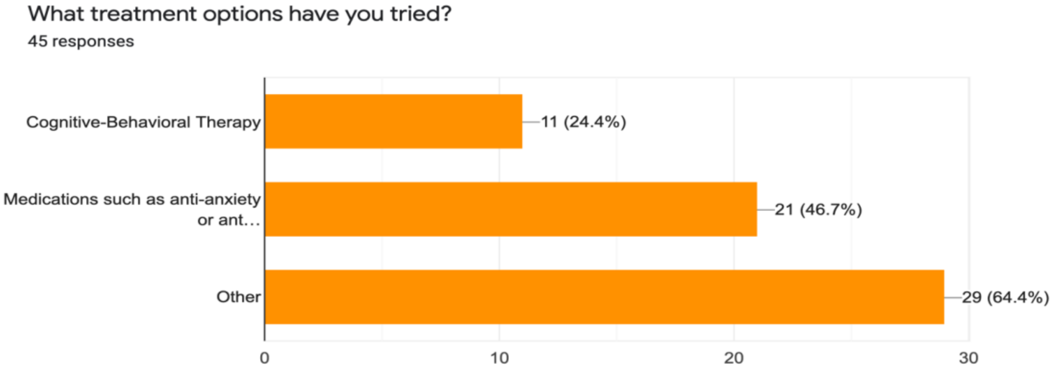

Figure S4. Treatment strategies tried by individuals for Skin picking behavior.

About 24.4% (*n* = 11) of the 45 individuals reported having taken CBT and 46.7% (*n* = 21) reported using medications such as anti-anxiety or anti-depressants while 64.4% (*n* = 29) reported using other methods such band aids, gloves, bitter nail polish, fidget toys, moisturizers and antibiotic creams, rubber bands and keen bracelet.

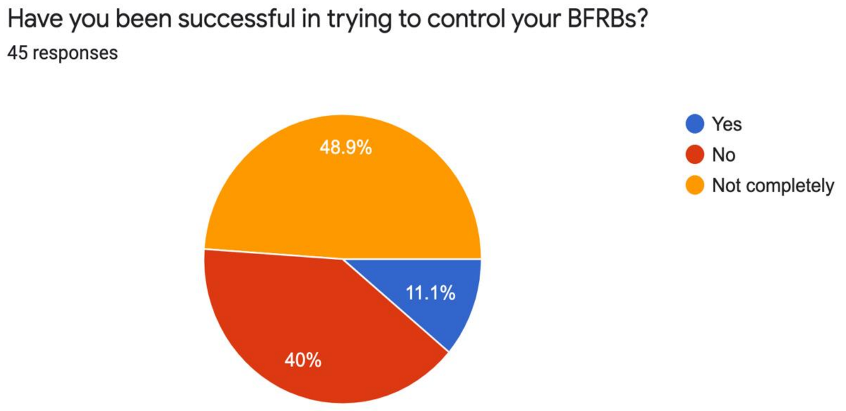

Figure S5. Success rate of controlling skin picking behavior.

Only 11.1% of 45 reported having being successful in controlling their BFRBs.

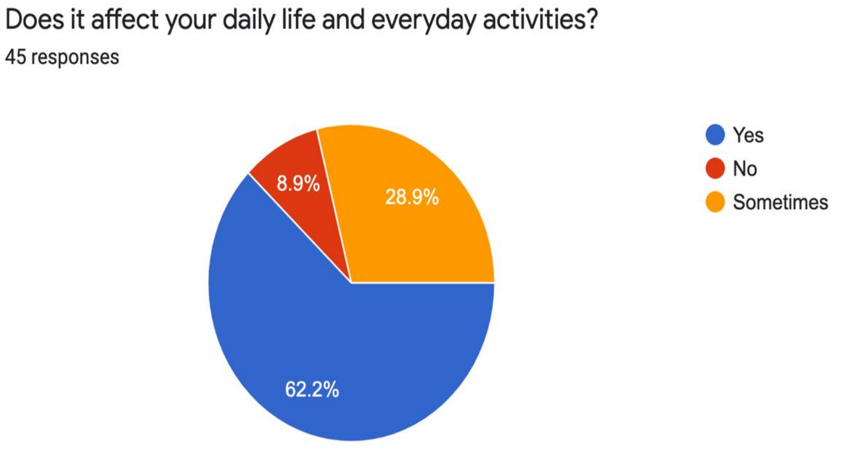

Figure S6. Interference of skin picking behavior on daily life of individuals.

About 62.2% reported that engaging in the behaviour interfere with their everyday life activities and 28.9% reported that it interferes sometimes.

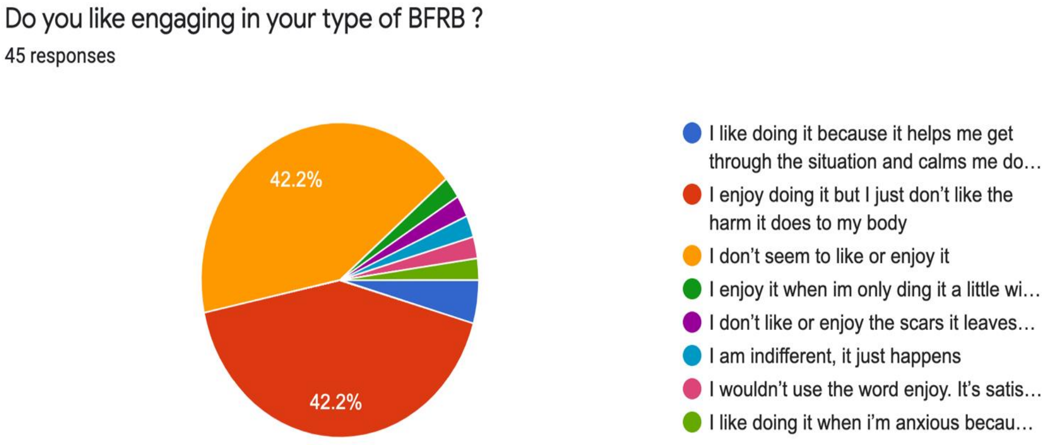

Figure S7. Interest in engaging in skin picking behavior.

About 42.2% reported that they enjoy doing it but just don't like the harm it does to their body while 42.2% reported that they don't seem to like or enjoy engaging in the behaviour.

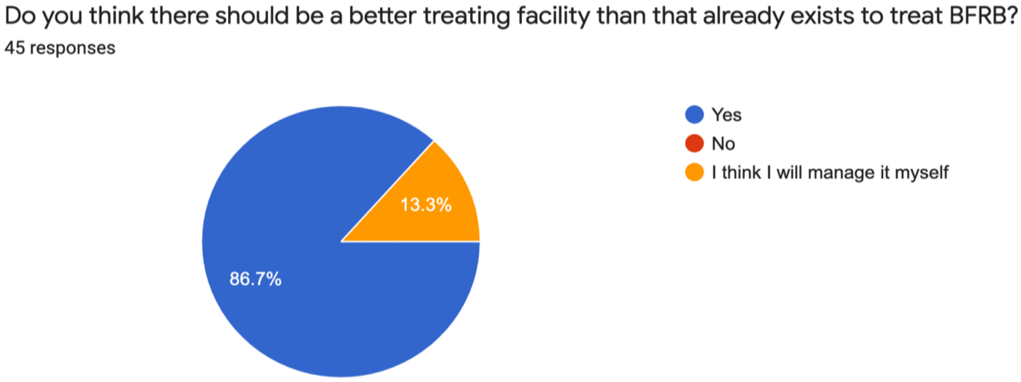

Figure S8. Need for better treating facility for BFRB.

About 86.7% of the individuals think that there should be better treating facility than that already exists for the treatment of BFRBs.

3. Conclusions

There is a need for alternative treatment strategy as the need is represented by 86.7% of the individuals with skin picking in this survey. It is very important to consider opinions of individuals with SPD when designing treatment strategies. In this survey that we conducted about 42.2% reported that they enjoy engaging in the behaviour but don't like the harm it does to their bodies. Another 42.2% reported that they don't seem to either like or enjoy engaging in skin picking behaviour (Figure S7). Considering these answers, response substitution and response prevention methods seemed to be more apt and which is why physical barrier strategies have been proposed and discussed in this review.

4. Limitations

It is a self-reported survey. Most of the individuals are not officially diagnosed by a psychiatrist. Survey was conducted to understand all body focussed repetitive behaviours and not just Dermatillomania.

Table S1. Pharmacological drug treatments for skin picking disorder (SPD).

Antidepressants					
**Drug**	**MOA**	**Type of Study**	**Result**	**Follow up/Relapse**	**Studies with no /Negative Effect**
Fluoxetine	SSRI	6-week open label test	20–40 mg/day, Skin picking reduced up to 60% by observer and self-reported scale in 8 out of 15 individuals [1]	Discontinuation caused recurrence	[2–4]
Sertraline	SSRI	Case report	*Partial improvement in psychogenic excoriation in one individual [5]	No data	[6–8]
Fluvoxamine	SSRI	12-week open label trial	7 completers out of 14 participants showed improvement on YBOCS scale and significant reduction in skin picking [9]	No data	[10]
Trazodone	SARI	Case report	Not effective at all (50 mg at night for 6 months) [11]	No data	[11]
Paroxetine	SSRI	Case report	40mg/day for 4months – skin picking completely stopped [11]	No skin picking for one year	[10]
Citalopram	SSRI	Case reports	No reduction or improvement in skin picking [5,12]	-	[5,12]
Venlafaxine	SNRI	Case Report	Skin picking behavior was aggravated [5]	-	[5]
Doxepin	SNRI	Case Report	Symptomatic and clinical improvement in neurotic excoriation in two individuals [13]	Effects Maintained even after two months [14]	-
		Case Report	Doxepin + HRT was helpful [14]		
Escitalopram	SSRI	18-week open label trial	Mean maximally tolerated dose of 25mg/day. 57.9% of 19 completers (out of 29 participants) showed >25% decrease in SPS score with a CGI score of <3 [15]	-	-
		Case Report	10 mg/day in combination with behavioural therapy showed reduction in picking from 20/24 to 0/24 on SPS [14]	-	
		Case Report	10 mg/day resulted in significant reduction in skin picking behavior in 2 weeks [16]	Recurrence of skin picking behavior on discontinuation [16]	
Agomelatine (MT_1MT_2 receptor agonist)	5-HT2C Antagonist	Case report	25 mg/day Agomelatine in augmentation with sertraline and quetiapine reduced skin picking [17]	Effects maintained at 3 and 6 months follow up after discontinuation	-

Antipsychotics					
**Drug**	**MOA[18]**	**Type of Study**	**Result**	**Follow up/Relapse**	**Studies with no /Negative Effect**
Clozapine	D2 & 5HT2A receptor antagonism	Case Report	300–400 mg/day resulted in complete disappearance of excoriation disorder [19]	-	[16]
Olanzapine	D2 & 5HT2A receptor antagonism	Case Report	2.5 mg at bedtime for 4 weeks resulted in a significant decline in skin picking behavior [20]	No recurrence of skin picking even after 4 months after discontinuation[20]	-
		Case Series	2.5–10 mg/daily showed rapid improvement in skin picking behavior in 6 individuals [5]	Improvements persisted over 3 and 6 months follow ups in two individuals [5]	
Aripiprazole	Agonism of D2 & 5-HT1A receptor, 5-HT2A receptor antagonist	Case Report	10 mg/day Aripiprazole with 375 mg/day venlafaxine reduced excoriations [21]	Benefit was sustained at 6 months [21]	-
		Case Report	10 mg/day Aripiprazole with 225 mg/day Venlafaxine reduced skin picking desire rapidly [22]	No skin picking even after 3 weeks follow up [22]	
		Case Report	2.5 mg/day Aripiprazole with 100mg/day fluoxetine resulted in completed resolution of skin picking in two weeks [23]	-	
Paliperidone	D2 & 5HT2A receptor antagonism	Case Report	6 mg/day Paliperidone with 80 mg/day Fluoxetine resulted in a decrease in SPS score from 14 to 2 in 2 months [24]	Drugs taken for 9 months and still continues to be in remission from skin picking [24]	-
Risperidone	D2 & 5HT2A receptor antagonism	Case Report	1 mg/day to 2 mg/day Risperidone with HRT and CBT reduced skin picking time, frequency and intensity [25]	-	[26]
		Case Report	0.5mg to 1.5 mg/day Risperidone for 4 weeks reduced skin picking [27]	Complete cessation of skin picking at 6-week follow-up [27]	
		Case Report	Risperidone with Citalopram given for a month reduced methylphenidate induced skin picking [28]	No Recurrence at subsequent follow up [28]	
Haloperidol	Dopamine D2 receptor antagonism	Case Report	0.7 mg/day Haloperidol with 300 mg/day Fluvoxamine for 10weeks reduced skin picking tendency [29]	-	-
Quetiapine	D2 & 5HT2A receptor antagonism	Case Report	Up to 200 mg/day Quetiapine with up to 150 mg/day Sertraline for 25 days showed no effect on skin picking [17]	-	[17]
**Opiod Antagonists**					
**Drug**	**MOA**	**Type of Study**	**Result**	**Follow up/Relapse**	**Studies with no /Negative Effect**
Naltrexone	Binds to opioid receptors in the CNS and blocks the effects of endogenous opioids	Case Report	50 mg/day reduced skin picking behavior [2]	Discontinuation caused recurrence of skin picking [2]	-
		Case Report	50 mg/day Naltrexone for 4 weeks along with 5mg/day Risperidone completely stopped skin picking behavior [7]	Recurrence of skin picking behavior when Naltrexone was stopped. Improved or no skin picking on	

				this combination for 15 months [7]	
**Anti-Epileptics**					
**Drug**	**MOA**	**Type of Study**	**Result**	**Follow up/Relapse**	**Studies with no /Negative Effect**
Lorazepam	Act on the limbic system and facilitate inhibitory effect of GABA	Case Report	3 mg/day for two years had no effect on the picking behavior [11]	-	[11]
Topiramate	Increases GABA activity and inhibiting glutamate activity- blocks neuronal excitability	12-week Open Label Trial<br><br>12-week open label pilot study	Up to 200 mg/day in two individuals was not at all effective [26]<br><br>25–200 mg/day improved skin picking time from 85 min to 30 min per day. 70% of 10 individuals with SPD showed much improved and very much improved on CGI-I. Scores on SPIS and SPS-Y-BOCS also improved [30]	-	-
Lamotrigine	Suppress the release of excitatory amino acid, glutamate	12-week open label trial<br><br>12-week randomized, double-blind, placebo-controlled trial [32]	25 mg to 300 mg/day showed significant reduction in two-thirds (of the 24 individuals with SPD) in time spent on skin picking and was considered much or very much improved [31]<br><br>12.5 mg to 300 mg/day. 32 individuals with SPD were divided into placebo and lamotrigine group. 7 individuals responded to lamotrigine while 5 individuals responded to placebo. Lamotrigine not very efficacious in individuals with SPD [32]	-	[32]
**Others**					
**ADHD drugs**					
**Drug**	**MOA**	**Type of Study**	**Result**	**Follow up/Relapse**	**Studies with no /Negative Effect**
Methylphenidate	Norepinephrine and dopamine reuptake inhibitor (NDRI)	Case Report<br><br>Case Report	30mg/day- reduced skin picking [33]<br><br>10mg/day modified release methylphenidate stopped skin picking [34]	-<br><br>No skin picking even after 2 months follow up [34]	[28]
Guanfacine extended release	-	Study	*Mean stable dosage of 2.75 mg/day resulted in 81.8% of 11 individuals with Prader-Willi syndrome showing improved skin behavior [35]	-	-
**Mood Stabilizer**					
Lithium Carbonate	Modulation of glutamate receptors	Case Report	300 mg to 900 mg/day showed improvement in skin picking behavior and acne excoriee [36]	-	-
**Others**					
N-acetyl cysteine	Converted to cysteine which is a substrate for cystine-	Open label pilot study	450 mg to 1200 mg/day in 35 individuals with PWS improved skin picking behavior. 71% of them showed complete resolution of skin picking behavior [37]	-	-

	glutamate antiporter and increases glutamate release into extracellular space	12-week randomized double-blind trial	1200 mg to 3000 mg/day showed improvements in NE-YBOCS and CGI severity score. 47% of the individuals were much or very much improved compared to 19% with Placebo [38]		
		Several case reports	Reduced or complete cessation of skin picking behavior [8,39–42]		
Inositol	Increase serotonin receptor sensitivity. Increase in GABA release	Case report	18 g/day Inositol with 40 mg/day Citalopram resulted in reduced skin picking and was considered very much improved [12]	-	-
Riluzole	Glutamate antagonist. Inhibits glutamate release thereby activating glutamate reuptake	Case Report	100 mg twice daily with 40 mg twice daily of fluoxetine decreased SPIS score in a month [43]	-	-

References

1. Bloch, M.R.; Elliott, M.; Thompson, H.; Koran, L.M. Fluoxetine in pathologic skin-picking: Open-label and double-blind results. *Psychosomatics* **2001**, *42*, 314–319. doi:10.1176/appi.psy.42.4.314.
2. Benjamin, E.; Buot-Smith, T. Naltrexone and fluoxetine in Prader-Willi syndrome. *J. Am. Acad. Child. Adolesc. Psychiatry* **1993**, *32*, 870–873. doi:10.1097/00004583-199307000-00025.
3. Schepis, C.; Failla, P.; Siragusa, M.; Palazzo, R.; Romano, C. Failure of fluoxetine to modify the skin-picking behaviour of Prader-Willi syndrome. *Australas. J. Dermatol.* **1998**, *39*, 57–58. doi:10.1111/j.1440-0960.1998.tb01247.x.
4. Çoşkun, F.; Bilgiç, A. Fluoxetine-Induced Skin Picking and Compulsive Behaviors in a Preschool Girl. *Clin. Neuropharmacol.* **2018**, *41*, 192–193. doi:10.1097/WNF.0000000000000298.
5. Blanch, J.; Grimalt, F.; Massana, G.; Navarro, V. Efficacy of olanzapine in the treatment of psychogenic excoriation. *Br. J. Dermatol.* **2004**, *151*, 714–716. doi:10.1111/j.1365-2133.2004.06151.x.
6. O'Sullivan, R.L.; Phillips, K.A.; Keuthen, N.J.; Wilhelm, S. Near-fatal skin picking from delusional body dysmorphic disorder responsive to fluvoxamine. *Psychosomatics* **1999**, *40*, 79–81. doi:10.1016/S0033-3182(99)71276-4.
7. Banga, A.; Connor, D.F. Effectiveness of naltrexone for treating pathologic skin picking behavior in an adolescent with Prader-Willi syndrome. *J. Child. Adolesc. Psychopharmacol*. **2012**, *22*, 396–398. doi:10.1089/cap.2012.0028.
8. Silva-Netto, R.; Jesus, G.; Nogueira, M.; Tavares, H. N-acetylcysteine in the treatment of skin-picking disorder. *Braz. J. Psychiatry*. **2014**, *36*, 101. doi:10.1590/1516-4446-2013-1154.
9. Arnold, L.M.; Mutasim, D.F.; Dwight, M.M.; Lamerson, C.L.; Morris, E.M.; McElroy, S.L. An open clinical trial of fluvoxamine treatment of psychogenic excoriation. *J. Clin. Psychopharmacol.* **1999**, *19*, 15–18. doi:10.1097/00004714-199902000-00005.
10. Denys, D.; van Megen, H.J.G.M.; Westenberg, H.G.M. Emerging skin-picking behaviour after serotonin reuptake inhibitor-treatment in patients with obsessive-compulsive disorder: Possible mechanisms and implications for clinical care. *J. Psychopharmacol.* **2003**, *17*, 127–129. doi:10.1177/0269881103017001718.
11. Ravindran, A.V.; Lapierre, Y.D.; Anisman, H. Obsessive-compulsive spectrum disorders: Effective treatment with paroxetine. *Can. J. Psychiatry* **1999**, *44*, 805–807. doi:10.1177/070674379904400808.
12. Seedat, S.; Stein, D.J.; Harvey, B.H. Inositol in the treatment of trichotillomania and compulsive skin picking. *J. Clin. Psychiatry* **2001**, *62*, 60–61. doi:10.4088/jcp.v62n0112f.
13. Harris, B.A.; Sherertz, E.F.; Flowers, F.P. Improvement of chronic neurotic excoriations with oral doxepin therapy. *Int. J. Dermatol.* **1987**, *26*, 541–543. doi:10.1111/j.1365-4362.1987.tb02300.x.
14. Nirmal, B.; Shenoi, S.D.; Rai, S.; Sreejayan, K.; Savitha, S. "Look beyond skin": Psychogenic excoriation—a series of five cases, *Indian J. Dermatol.* **2013**, *58*, 246. doi:10.4103/0019-5154.110885.
15. Keuthen, N.J.; Jameson, M.; Loh, R.; Deckersbach, T.; Wilhelm, S.; Dougherty, D.D. Open-label escitalopram treatment for pathological skin picking. *Int. Clin. Psychopharmacol*. **2007**, *22*, 268–274. doi:10.1097/YIC.0b013e32809913b6.
16. Reddy, B.; Das, S.; Guruprasad, S. A case of clozapine-induced skin picking behavior. *Gen. Psychiatr*. **2018**, *31*, e000012. doi:10.1136/gpsych-2018-000012.
17. Antoniadis, D.; Floros, G.D. Nikolaidis, N.; Garyfallos, G. Response to agomelatine: Treatment of an obsessive skin picking episode. *Ann. Clin. Psychiatry* **2013**, *25*, 228–229.

18. Drug Bank. Available Online: https://www.drugbank.ca/ (accessed on June 2020)
19. Calandrella, D.; Rizzi, M.; Ferré, F.M.; Romito, L.M. Excoriation disorder as a risk factor for deep brain stimulation hardware removal. *J. Neurol. Sci.* **2017**, *373*, 342–343. doi:10.1016/j.jns.2017.01.034.
20. Gupta, M.A.; Gupta, A.K. Olanzapine may be an effective adjunctive therapy in the management of acne excoriée: A case report. *J. Cutan. Med. Surg.* **2001**, *5*, 25–27. doi:10.1177/120347540100500106.
21. Carter, W.G.; Shillcutt, S.D. Aripiprazole augmentation of venlafaxine in the treatment of psychogenic excoriation. *J. Clin. Psychiatry* **2006**, *67*, 1311. doi:10.4088/jcp.v67n0821f.
22. Turner, G.A.; Sutton, S.; Sharma, A. Augmentation of Venlafaxine with Aripiprazole in a Case of Treatment-resistant Excoriation Disorder. *Innov Clin Neurosci.* **2014**, *11*, 29–31.
23. Curtis, A.R.; Richards, R.W. The treatment of psychogenic excoriation and obsessive compulsive disorder using aripiprazole and fluoxetine. *Ann Clin Psychiatry* **2007**, *19*, 199–200. doi:10.1080/10401230701465277.
24. Spiegel, D.R.; Finklea, L. The recognition and treatment of pathological skin picking: a potential neurobiological underpinning of the efficacy of pharmacotherapy in impulse control disorders. *Psychiatry (Edgmont)* **2009**, *6*, 38–42.
25. Okan Ibiloğlu, A.; Atli, A.; Kaya, M.C.; Demir, S.; Bulut, M.; Sir, A. A Case of Skin Picking Disorder of a Patient with a History of Childhood Abuse. *Noro Psikiyatr. Ars.* **2016**, *53*, 181–183. doi:10.5152/npa.2015.10110.
26. Jafferany, M.; Shireen, F. Ibrahim, A. An open-label trial of topiramate in the treatment of skin picking in pervasive developmental disorder not otherwise specified. *Prim. Care Companion. J. Clin. Psychiatry* **2010**, doi:10.4088/PCC.09l00829yel.
27. Roi, C.; Bazzano, A. Improvement in Excoriation (Skin-Picking) with use of Risperidone in a Patient with Developmental Disability. *Pediatr. Rep.* **2017**, *9*, 6946. doi:10.4081/pr.2017.6946.
28. Kara, T.; Akaltun, İ. Newly Developed Skin Picking After Methylphenidate Treatment in Attention Deficit Hyperactivity Disorder: Possible Mechanisms. *Clin. Neuropharmacol.* **2018**, *41*, 28–30. doi:10.1097/WNF.0000000000000262.
29. Luca, M.; Vecchio, C.; Luca, A.; Calandra, C. Haloperidol augmentation of fluvoxamine in skin picking disorder: A case report. *J. Med. Case Rep.* **2012**, *6*, 219. doi:10.1186/1752-1947-6-219.
30. Jafferany, M.; Osuagwu, F.C. Use of Topiramate in Skin-Picking Disorder: A Pilot Study. *Prim. Care Companion. CNS Disord.* **2017**, *19*. (2017). doi:10.4088/PCC.16m01961.
31. Grant, J.E.; Odlaug, B.L.; Kim, S.W. Lamotrigine treatment of pathologic skin picking: An open-label study. *J. Clin. Psychiatry* **2007**, *68*, 1384–1391. doi:10.4088/jcp.v68n0909.
32. Grant, J.E.; Odlaug, B.L.; Chamberlain, S.R.; Kim, S.W. A double-blind, placebo-controlled trial of lamotrigine for pathological skin picking: treatment efficacy and neurocognitive predictors of response. *J. Clin. Psychopharmacol.* **2010**, *30*, 396–403. doi:10.1097/JCP.0b013e3181e617a1.
33. Bernardes, C.; Mattos, P.; Nazar, B.P. Skin picking disorder comorbid with ADHD successfully treated with methylphenidate. *Braz. J. Psychiatry*. **2018**, *40*, 111. doi:10.1590/1516-4446-2017-2395.
34. Çolak Sivri, R.; Çolak, B. Cessation of Skin Picking Symptoms with Methylphenidate Treatment in a Child With Comorbid Skin Picking and Attention-Deficit/Hyperactivity Disorder, *Clin Neuropharmacol.* **2019**, *42*, 105–107. doi:10.1097/WNF.0000000000000338.
35. Singh, D.; Wakimoto, Y.; Filangieri, C.; Pinkhasov, C.; Angulo, M. Guanfacine Extended Release for the Reduction of Aggression, Attention-Deficit/Hyperactivity Disorder Symptoms, and Self-Injurious Behavior in Prader-Willi Syndrome—A Retrospective Cohort Study. *J. Child. Adolesc. Psychopharmacol.* **2019**, *29*, 313–317. doi:10.1089/cap.2018.0102.
36. Gupta, M.A. Emotional regulation, dissociation, and the self-induced dermatoses: Clinical features and implications for treatment with mood stabilizers. *Clin. Dermatol.* **2013**, *31*, 110–117. doi:10.1016/j.clindermatol.2011.11.015.
37. Miller, J.L.; Angulo, M. An open-label pilot study of N-acetylcysteine for skin-picking in Prader-Willi syndrome. *Am. J. Med. Genet. A.* **2014**, *164A*, 421–424. doi:10.1002/ajmg.a.36306.
38. Grant, J.E.; Chamberlain, S.R.; Redden, S.A.; Leppink, E.W.; Odlaug, B.L. Kim, S.W. N-Acetylcysteine in the Treatment of Excoriation Disorder: A Randomized Clinical Trial. *JAMA Psychiatry* **2016**, *73*, 490–496. doi:10.1001/jamapsychiatry.2016.0060.
39. Odlaug, B.L.; Grant, J.E. N-acetyl cysteine in the treatment of grooming disorders. *J. Clin. Psychopharmacol.* **2007**, *27*, 227–229. doi:10.1097/01.jcp.0000264976.86990.00.
40. Grant, J.E.; Odlaug, B.L.; Chamberlain, S.R.; Keuthen, N.J.; Lochner, C.; Stein, D.J. Skin picking disorder. *Am. J. Psychiatry* **2012**, *169*, 1143–1149. doi:10.1176/appi.ajp.2012.12040508.
41. Percinel, I.; Yazici, K.U. Glutamatergic dysfunction in skin-picking disorder: treatment of a pediatric patient with N-acetylcysteine. *J. Clin Psychopharmacol.* **2014**, *34*, 772–774. doi:10.1097/JCP.0000000000000210.
42. Kiliç, F.; Keleş, S. Repetitive Behaviors Treated With N-Acetylcysteine: Case Series. *Clin. Neuropharmacol.* **2019**, *42*, 139–141. doi:10.1097/WNF.0000000000000352.
43. Sasso, D.A.; Kalanithi, P.S.A.; Trueblood, K.V.; Pittenger, C.; Kelmendi, B.; Wayslink, S.; Malison, R.T.; Krystal, J.H.; Coric, V. Beneficial effects of the glutamate-modulating agent riluzole on disordered eating and pathological skin-picking behaviors. *J. Clin. Psychopharmacol.* **2006**, *26*, 685–687. doi:10.1097/01.jcp.0000245567.29531.d6.
